# Seroprevalence of *Toxoplasma gondii* in domestic pigs, sheep, cattle, wild boars, and moose in the Nordic-Baltic region: A systematic review and meta-analysis

**DOI:** 10.1016/j.parepi.2019.e00100

**Published:** 2019-03-04

**Authors:** Abbey Olsen, Rebecca Berg, Maarja Tagel, Kärt Must, Gunita Deksne, Heidi Larsen Enemark, Lis Alban, Maria Vang Johansen, Henrik Vedel Nielsen, Marianne Sandberg, Anna Lundén, Christen Rune Stensvold, Sara M. Pires, Pikka Jokelainen

**Affiliations:** a Section for Parasitology and Aquatic Pathobiology, Department of Veterinary and Animal Sciences, Faculty of Health and Medical Sciences, University of Copenhagen, Dyrlægevej 100, DK-1870 Frederiksberg C, Denmark; b Department of Food Safety, Veterinary Issues & Risk Analysis, Danish Agriculture & Food Council, Axelborg, Axeltorv 3, DK-1609 Copenhagen, Denmark; c Section for Organismal Biology, Faculty of Science, University of Copenhagen, Thorvaldsensvej 40, DK-1871 Frederiksberg, Denmark; d Institute of Veterinary Medicine and Animal Sciences, Estonian University of Life Sciences, Kreutzwaldi 62, 51006 Tartu, Estonia; e Department of Parasitology, Institute of Food Safety, Animal Health and Environment “BIOR”, Lejupes Str. 3, LV-1076 Riga, Latvia; f Department of Zoology and Animal Ecology, Faculty of Biology, University of Latvia, Jelgavas Str. 1, LV-1004 Riga, Latvia; g Norwegian Veterinary Institute, Department of Animal Health and Food Safety, P.O. Box 750, Sentrum, NO-0106 Oslo, Norway; h Laboratory of Parasitology, Department of Bacteria, Parasites & Fungi, Infectious Disease Preparedness, Statens Serum Institut, Artillerivej 5, 2300 Copenhagen S, Denmark; i National Veterinary Institute, Department of Microbiology, SE-751 89 Uppsala, Sweden; j National Food Institute, Technical University of Denmark, Kemitorvet 201, 2800 Kgs. Lyngby, Denmark; k Faculty of Veterinary Medicine, University of Helsinki, P.O. Box 66, 00014 Helsinki, Finland

**Keywords:** Food-borne, Meat-borne, Europe, Seroepidemiology, Toxoplasmosis, Zoonosis

## Abstract

**Background:**

*Toxoplasma gondii* is an important foodborne zoonotic parasite. Meat of infected animals is presumed to constitute a major source of human infection and may be a driver of geographical variation in the prevalence of anti-*T. gondii* antibodies in humans, which is substantial in the Nordic-Baltic region in northern Europe. However, data on seroprevalence of *T. gondii* in different animal species used for human consumption are scattered.

**Methods:**

We conducted a systematic review of seroprevalence studies and meta-analysis to estimate the seroprevalence of *T. gondii* in five animal species that are raised or hunted for human consumption in the Nordic-Baltic region: domestic pigs (*Sus scrofa domesticus*), sheep (*Ovis aries*), cattle (*Bos taurus*), wild boars (*Sus scrofa*), and moose (*Alces alces*). We searched for studies that were conducted between January 1990 and June 2018, and reported in articles, theses, conference abstracts and proceedings, and manuscripts. Subgroup analyses were performed to identify variables influencing the seroprevalence.

**Findings:**

From a total of 271 studies identified in the systematic review, 32 were included in the meta-analysis. These comprised of 13 studies on domestic pigs, six on sheep, three on cattle, six on wild boars, and four on moose. The estimated pooled seroprevalence of *T. gondii* was 6% in domestic pigs (CI_95%_: 3–10%), 23% in sheep (CI_95%_: 12–36%), 7% in cattle (CI_95%_: 1–21%), 33% in wild boars (CI_95%_: 26–41%), and 16% in moose (CI_95%_: 10–23%). High heterogeneity was observed in the seroprevalence data within each species. In all host species except wild boars, the pooled seroprevalence estimates were significantly higher in animals >1 year of age than in younger animals. Not all studies provided information on animal age, sensitivity and specificity of the serological method employed, and the cut-off values used for defining an animal seropositive.

**Conclusions:**

A substantial proportion of animals raised or hunted for human consumption in the region had tested positive for *T. gondii*. This indicates widespread exposure to *T. gondii* among animals raised or hunted for human consumption in the region. Large variations were observed in the seroprevalence estimates between the studies in the region; however, studies were too few to identify spatial patterns at country-level.

## 1 Introduction

*Toxoplasma gondii* is a zoonotic protozoan parasite distributed worldwide and globally ranked fourth among food-borne parasites that pose a threat to public health (FAO/WHO, Food and Agriculture Organization/World Health Organization, 2014). *Toxoplasma gondii* has a complex life cycle where felids are the only known definitive hosts (Dubey, 2009a; Dubey, 2010). Infected felids can shed millions of oocysts in their feces for a limited time. After sporulation in the environment, the oocysts can infect a wide range of hosts through contaminated soil, water, feed, and food (Dubey, 2010; Jones and Dubey, 2012). In the infected hosts, the parasite undergoes asexual multiplication and can form tissue cysts. *Toxoplasma gondii* tissue cysts in the meat of animals raised or hunted for human consumption pose a risk to humans if consumed raw or without thorough cooking (Jones and Dubey, 2012). Among the many possible routes of *T. gondii* infection, ingestion of viable parasites in the tissue cysts of meat originating from infected animals is considered important in humans (Cook et al., 2000; Tenter, 2009).

In most animal host species, the majority of *T. gondii* infections are subclinical. In farm animals, abortions are considered the most relevant clinical manifestation, especially in sheep, and may lead to economic losses (Dubey, 2009b; Dubey, 2009c; Dubey, 2010). In humans, the infection is often asymptomatic or causes mild symptoms. However, the infection may result in ocular toxoplasmosis; in pregnant women, the infection may result in congenital toxoplasmosis; and especially in immunosuppressed individuals, the infection may be fatal (Montoya and Liesenfeld, 2004). Additionally, recent studies have reported associations between *T. gondii* infection and psychiatric disorders (Sutterland et al., 2015). The disease burden caused by *T. gondii* has recently gained more attention (Torgerson and Mastroiacovo, 2013; Mangen et al., 2015; Nissen et al., 2017).

*Toxoplasma gondii* cannot be detected by routine meat inspection, and there has been relatively little emphasis on the prevention of *T. gondii* infection in the food chain. Seroepidemiological studies have shown that both farm animals raised for human consumption and game animals farmed or hunted for human consumption are commonly exposed to *T. gondii* worldwide (Dubey, 2010; Opsteegh et al., 2016).

In the Nordic-Baltic region, the *T. gondii* seroprevalence in humans varies markedly between the countries. It has been reported as 9% in pregnant women in Norway (Findal et al., 2015), 10% in individuals aged 20–44 years in Iceland (Birgisdóttir et al., 2006), 15% in veterinarians in Finland (Siponen et al., 2019), 20% in individuals aged ≥30 years in Finland (Suvisaari et al., 2017), 20% in pregnant women in Finland (Lappalainen et al., 1992), 23% in individuals aged 20–44 years in Sweden (Birgisdóttir et al., 2006), 28% in pregnant women in Denmark (Lebech et al., 1994), 38% in children aged 14–18 years in Estonia (Lassen et al., 2016), 55% in individuals aged 20–44 years in Estonia (Birgisdóttir et al., 2006), 56% in the general adult population in Estonia (Lassen et al., 2016), and 62% in individuals tested at a clinic in Estonia (Pehk, 1994). These geographical differences in *T. gondii* seroprevalence may reflect differences in food consumption habits between countries, geographic variation in *T. gondii* prevalence in animals raised or hunted locally for human consumption, or different levels of oocyst contamination of the environment across the region. Quantification of the seroprevalence of *T. gondii* in animals used for human consumption can help estimate the infection risk for humans from different types of meat; however, only sporadic data are available. Therefore, we performed a systematic review and meta-analysis to estimate the seroprevalence of *T. gondii* in selected animals raised or hunted for human consumption across the Nordic-Baltic region.

## 2 Materials and methods

We performed a systematic review of seroprevalence studies on *T. gondii* in five animal species in the Nordic-Baltic region. The study was carried out following the recommendations given in “Preferred Reporting Items for Systematic review and Meta-Analysis” (PRISMA) (Liberati et al., 2009). Seroprevalence was defined as the proportion of animals that tested positive out of the total number of animals tested.

### 2.1 Search strategy and data sources

We set out to identify all studies reporting *T. gondii* seroprevalence in domestic pigs (*Sus scrofa domesticus*), sheep (*Ovis aries*), cattle (*Bos taurus*), wild boars (*Sus scrofa*), and moose (*Alces alces*) from the region comprising the Nordic countries (Denmark including the Faroe Islands and Greenland, Finland including the Åland Islands, Iceland, Norway, and Sweden) and the Baltic countries (Estonia, Latvia, and Lithuania). Studies published from beginning of January 1990 to the end of June 2018, and manuscripts identified by the end of June 2018, were considered. Studies reported in peer-reviewed articles, theses, conference abstracts and proceedings, and scientific article manuscripts, in all languages, were considered eligible.

Two of the authors (AO, RB) independently searched for peer-reviewed articles from the CAB Abstract and the MedLine database, using relevant keywords with Boolean operators ‘OR’ and ‘AND’ (Appendix A, Table A.1). Additionally, one author (AO) searched the ProQuest database (Appendix A, Table A.2), the Danish national research database (www.forskningsdatabasen.dk), and the Bulletin of Scandinavian-Baltic Society for Parasitology (http://sbsp.eu/index.php/bulletin). The last search was done on the 30th of June 2018.

The corresponding authors of the identified eligible studies were contacted to identify further studies (Appendix A, Table A.3). Furthermore, the reference lists of the identified eligible studies were screened to identify further studies (particularly grey literature).

### 2.2 Study selection

Search results from the two databases and other sources were combined. Eligible studies were selected using inclusion and exclusion criteria (Table 1). Two of the authors (AO, RB) used these criteria to independently screen the title and the abstract of each study. Additionally, they identified duplicates and recorded the reason for exclusion of any study during the screening process. The data representing the eligible studies for data extraction were merged and managed in Microsoft Excel 2010 (Appendix C).

**Table 1:** Inclusion and exclusion criteria for the selection of eligible studies for a systematic review on *Toxoplasma gondii* seroprevalence in domestic pigs, sheep, cattle, wild boars and moose in the Nordic-Baltic region.

Inclusion criteria	Exclusion criteria
All studies (journal articles, theses, reports, scientific article manuscripts) on *T. gondii* seroprevalence published between 1990 and 2018 (except manuscripts, unpublished at inclusion)	Studies on *T. gondii* seroprevalence published before 1990
Samples (blood and meat juice) collected between 1990 and 2018	Samples collected before 1990
All languages	
Countries from the Nordic and Baltic region i.e., Denmark including the Faroe Islands and Greenland, Finland including the Åland Islands, Iceland, Norway, Sweden, Estonia, Latvia, and Lithuania	Countries outside the Nordic-Baltic region
Domestic pigs (*Sus scrofa domesticus*), sheep (*Ovis aries*), cattle (*Bos taurus*), wild boars (*Sus scrofa*) and moose (*Alces alces*)	All other host species
Seroprevalence studies conducted at animal-level	Herd-level seroprevalence studies
Cross-sectional, cohort and unstructured study designs to estimate *T. gondii* seroprevalence in apparently healthy animals Access to the full text of the study for data extraction	Experimental studies

### 2.3 Data extraction

A data extraction sheet was created in Microsoft Excel 2010 for the collection of the following information from the full-text publications: first author, year of publication, country, sample type (plasma, serum, meat juice), sampling period, host species, total number of animals sampled (per host species and per country), number of animals testing seropositive for *T. gondii*, total number of animals by age group (young, if ≤1 year; old, if >1 year), total number of seropositive animals by age group, the serological test used, the reported cut-off for classifying a sample as positive or negative, and the reported sensitivity and specificity of the serological test employed. Moreover, for domestic pigs, we collected the total number of pigs and total number of seropositive pigs by production system (indoor, outdoor). The data extraction sheet was pilot-tested by three of the authors (AO, MS, PJ) to assess the feasibility of filling it in with data for each host species and revised before the data extraction process. A data extraction group comprising of five of the authors was formed for each host species. These groups received the data extraction sheet along with a help file (Appendices D and E) and the assigned articles for reading. Each article was read by at least two authors. The extracted data were collated and compared, any disagreements in the results were resolved by discussion, and the final data were checked by three of the authors (AO, LA, PJ).

### 2.4 Data handling

When proportions approach zero or one, the variance of the proportions moves towards zero. As a result, studies with either high or low prevalence are given relatively high weight in the meta-analysis of prevalence (Barendregt et al., 2013). This is because the weight is calculated from the inverse of the variance of the prevalence estimate. Therefore, to avoid this, we transformed the prevalence data using the double arcsine method. Meta-analysis was then performed on the double arcsine transformed seroprevalence estimates. For reporting and interpretation of the results, the final pooled seroprevalence estimates and their 95% confidence intervals (CIs) were back-transformed to proportions to ease interpretation (Barendregt et al., 2013).

### 2.5 Evaluation of heterogeneity and pooled estimates

Meta-analysis was done for each host species separately. For each study, the seroprevalence and its 95% CI were calculated. Individual seroprevalence estimates were pooled using restricted maximum likelihood (REML) method with a random effects model. The individual seroprevalence estimates and the pooled seroprevalence estimates were first visually examined for heterogeneity using a forest plot. The studies were regarded homogenous, when the 95% CIs of all the seroprevalence estimates overlapped (Ried, 2006). Moreover, we used the inverse variance index (I^2^) to quantify heterogeneity, where I^2^ values of 25%, 50%, and 75% indicate low, moderate, and high heterogeneity, respectively (Higgins and Thompson, 2002).

To determine the effect of any influential studies on the overall pooled seroprevalence estimates for each host species, we conducted leave-one-out analyses (Appendix B, Figs. B.1 to B.5). A study was considered influential if the pooled seroprevalence estimate calculated without the study was not within the 95% CIs of the overall pooled seroprevalence estimate (Ding et al., 2017).

### 2.6 Subgroup analyses

The number of studies was insufficient for a multivariable regression analysis. Therefore, we performed subgroup analyses to identify possible sources of heterogeneity. The subgroups investigated were the two age groups, and for the studies on domestic pigs also the two production systems. The studies for which information about the subgroup was lacking were omitted from the analysis. Additionally, if a single study had data for both subgroups, then that study was considered as two separate studies in the subgroup analyses. The subgroup analyses were done for those host species for which at least three studies were available with data for the subgroup. Heterogeneity was explored by subgroups within each host species and leave-one-out analysis was conducted in each subgroup within each host species to detect influential studies (Appendix B, Figs. B.6 to B.15).

Subgroup analyses were performed using a mixed effect model, where the random effects model was used to pool the individual seroprevalence estimates within each subgroup and a fixed-effect of the variable of interest was used to test for significant differences between the subgroups (Borenstein et al., 2009; Wang, 2018). When computing the pooled seroprevalence estimates for each subgroup, we assumed the between-study variance to be the same for all subgroups, because the sample size within subgroups or one of the two subgroups was small (<5 studies).

The meta-analysis was performed in R studio (1.1.4), following the R script of Wang (2018).

## 3 Results

### 3.1 Search results and eligible studies

**Fig. 1:**
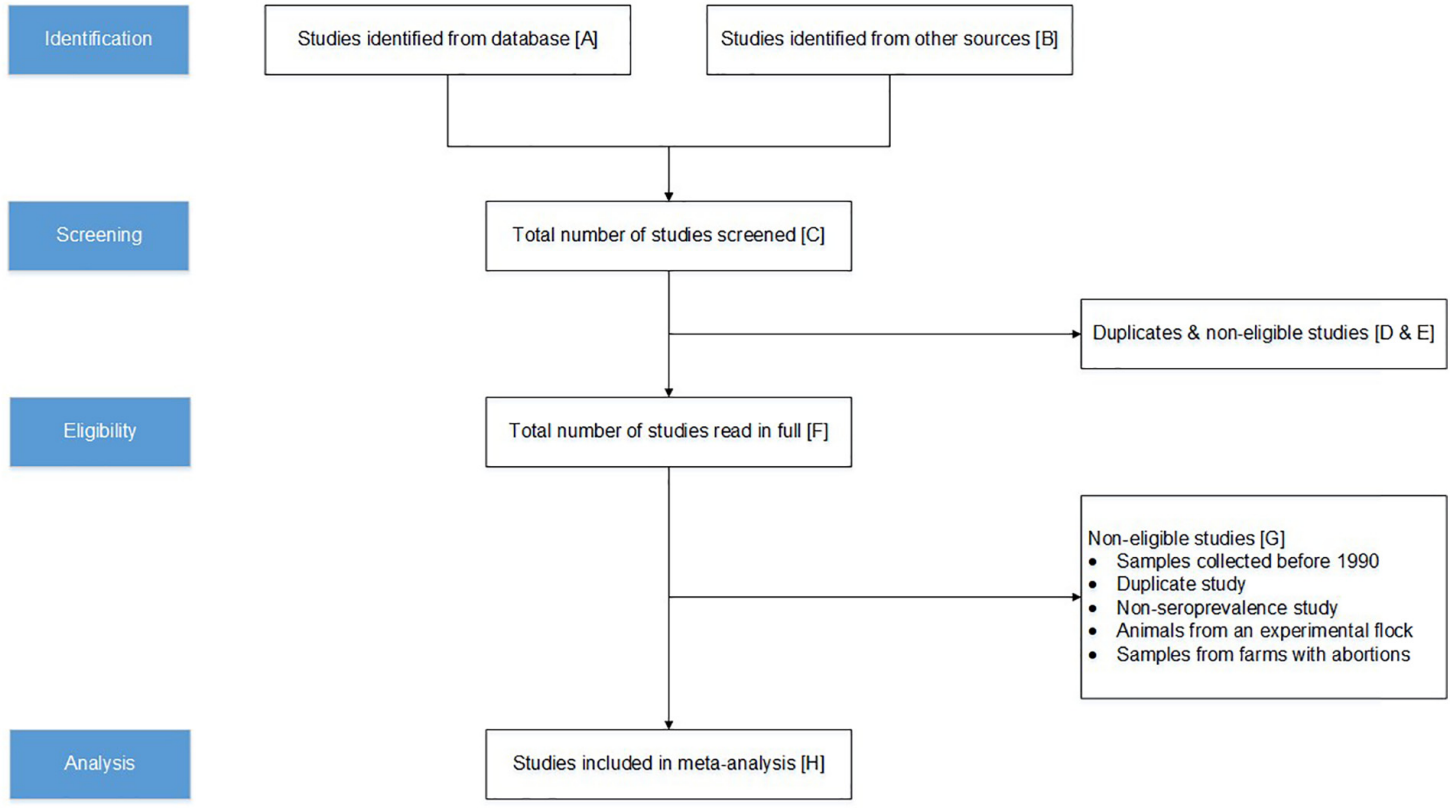
PRISMA flow-diagram shows the procedure for the selection of the eligible studies. The number of studies removed and selected at each step is marked with letters A–H and this information is displayed by host species in Table 2.

Fig. 1 outlines the PRISMA process followed for the systematic selection and removal of the studies for each of the five host species. In the literature search, a total of 271 studies were identified: 254 from the databases and 17 from other sources (Table 2). Altogether 11 of the 14 corresponding authors we contacted responded, yielding a response rate of 79% (Appendix A, Table A.3). From the 271 studies, 124 studies were duplicates and 105 studies did not meet the inclusion criteria of the screening process (Table 1). The remaining 42 studies were read in full, and 13 of them were further excluded as they did not meet the inclusion criteria. During the data extraction, the number of included studies increased with three because one of the studies on domestic pigs reported seroprevalence data also from a separate, unpublished study (Lind et al., 1994) and another study reported seroprevalence data from two studies on domestic pigs and two studies on sheep (Eglīte and Keidans, 2000) (Table 2). Therefore, a total of 32 studies (13 on domestic pigs, six on sheep, six on wild boars, four on moose, and three on cattle) were included in the meta-analysis.

**Table 2:** Overview of the number of studies selected and removed at each step of the PRISMA flow diagram for conducting a systematic review (Fig. 1).

	Identification		Screening			Eligibility		Meta-analysis
Host species	Database [A]	Other [B]	Total [C]	Duplicates [D]	Excluded [E]	Total [F]	Excluded [G]	Included [H]
Domestic pig	95	6	101	50	36	15	4	13^a,b^
Sheep	53	7	60	30	19	11	6	6^b^
Cattle	45	4	49	23	20	6	3	3
Wild boar	44	0	44	11	27	6	0	6
Moose	17	0	17	10	3	4	0	4
Total	254	17	271	124	105	42	13	32

a = Number of studies increased during the data extraction process. Lind et al. (1994) reported two studies on domestic pigs. b = Number of studies increased during the data extraction process. Eglīte and Keidans (2000) reported two studies on domestic pigs and two studies on sheep.

**Fig. 2:**
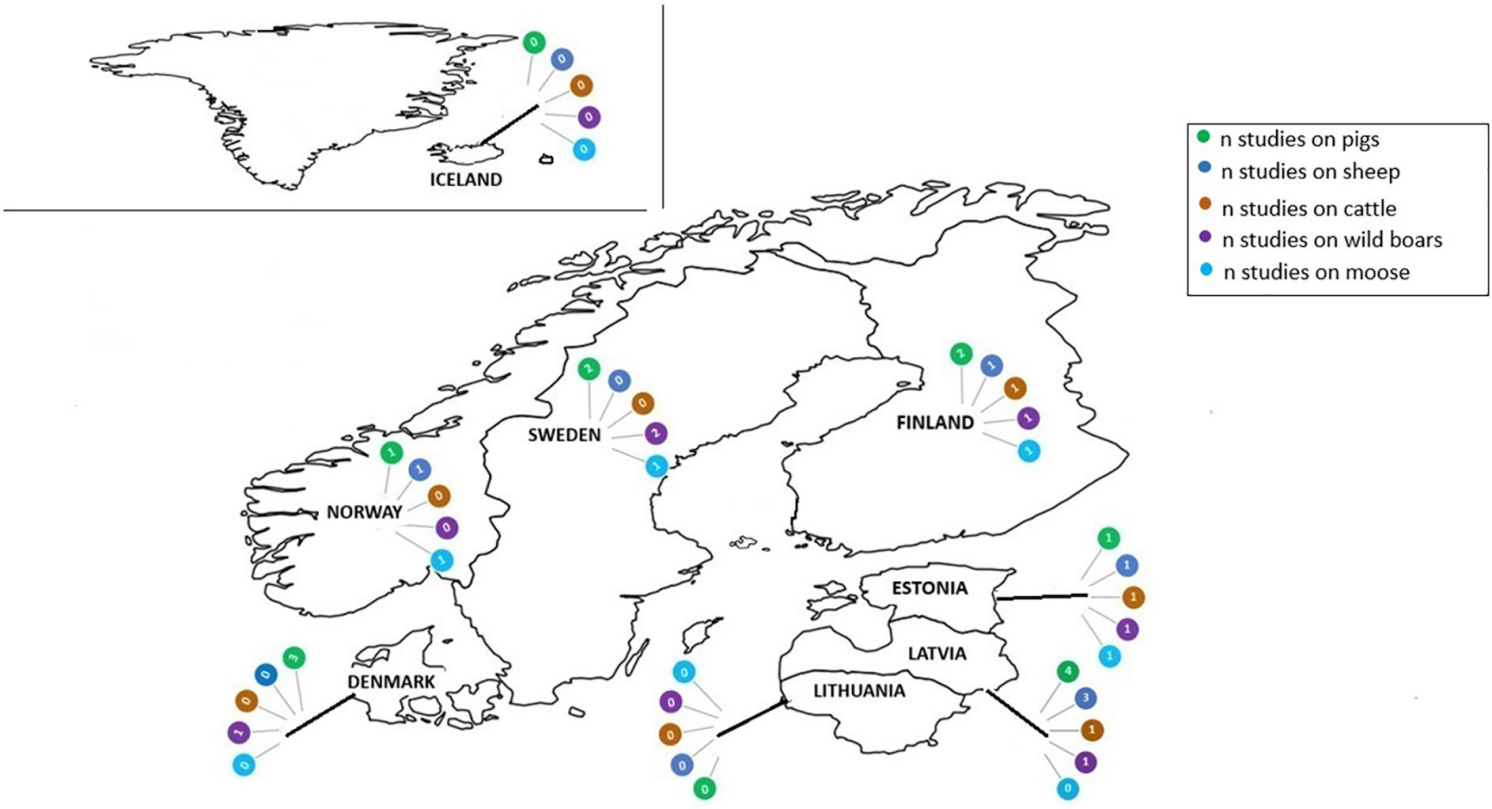
Number of *Toxoplasma gondii* seroprevalence studies in domestic pigs (*Sus scrofa domesticus*), sheep (*Ovis aries*), cattle (*Bos taurus*), wild boars (*Sus scrofa*), and moose (*Alces alces*), by country in the Nordic-Baltic region, 1990–2018.

The studies included are summarized in Table 3A, Table 3B, Table 3C, Table 3D, Table 3E. They were published between 1994 and 2018 and originated from six of the nine Nordic-Baltic countries. The number of studies per host species from each country ranged from zero to four (Fig. 2). The host species covered by the highest and lowest number of studies were domestic pigs (n = 13) and cattle (n = 3), respectively. The total number of animals tested was highest for domestic pigs (12,727), followed by sheep (6645), cattle (4445), wild boars (4237), and moose (2978).

**Table 3A:** Characteristics of the thirteen eligible studies on domestic pigs included in a meta-analysis for estimating seroprevalence of *Toxoplasma gondii* in the Nordic-Baltic region.

Authors	Year of publication	Country	Study period	Sample type	Serological method	Se^h^	Sp^i^	Total no. of pigs ≤1 year of age (no. of seropositives)	Total no. of pigs >1 year of age (no. of seropositives)	Total no. of pigs of unknown age (no. of seropositives)	Apparent seroprevalence (%)
Lind et al., 1994	1994	Denmark	1992–1993	Serum	ELISA^a^	0.94	0.92	n.r.	n.r.	4016 (124)	3.1
Lind et al., 1994	1994	Denmark	1992–1993	Serum	ELISA^a^	0.94	0.92	443 (26)	364 (70)	0	11.9
Skjerve et al., 1996	1996	Norway	1993–1994	Serum	ELISA^a^	n.r.	n.r.	n.r	n.r	1605 (42)	2.6
Eglīte and Keidans, 2000	2000	Latvia	1993–1998	Serum	CF^b^	n.r.	n.r.	n.r	n.r	265 (13)	4.9
Eglīte and Keidans, 2000	2000	Latvia	1998–2000	Serum	LA^c^	n.r.	n.r.	n.r	n.r	115 (35)	30.4
Lundén et al., 2002	2002	Sweden	1999–1999	Meat juice	ELISA^a^	0.94	0.92	695 (23)	110 (19)	2 (0)	5.2
Deksne, 2010	2010	Latvia	2010–2010	Meat juice	ELISA^a^	n.r.	n.r.	232 (16)	0	0	6.9
Deksne and Kirjušina, 2013	2013	Latvia	2010–2011	Meat juice	ELISA^a^	n.r.	n.r.	n.r	n.r	803 (34)	4.2
Felin et al., 2015	2015	Finland	2012–2013	Meat juice	ELISA^d^	0.99	0.93	425 (8)	928 (35)	0	3.2
Wallander et al., 2016	2016	Sweden	2011–2011	Meat juice	ELISA^e^	1.00	0.98	975 (55)	0	0	5.7
Kofoed et al., 2017	2017	Denmark	2016–2016	Serum	ELISA^a^	0.76	0.94	165 (8)	89 (30)	0	15.0
Santoro et al., 2017	2017	Estonia	2012–2012	Serum	DAT^f^	n.r.	n.r.	72 (2)	239 (19)	71 (1)	5.8
Felin et al., 2019	2019	Finland	2012–2014	Serum	ELISA^g^	1.00	1.00	1116 (8)	0	0	1.0

n.r. = not reported. a = In-house enzyme linked immunosorbent assay (ELISA). b = Unspecified commercial complement fixation test (CF). c = Latex agglutination test (LA). d = Commercial ELISA, Prio-CHECK Toxoplasma Ab Porcine test (Prionics AG,Schlieren-Zurich, Switzerland). e = Commercial ELISA, ID Screen Toxoplasmosis Indirect Multi-species IDvet Innovative Diagnostics Montpellier France). f = Commercial modified direct agglutination test (DAT), Toxo-Screen DA bioMérieux, Marcy-l'Étoile France [Cut-off, dilution of 1:40]. g = Commercial ELISA, pigtype® Toxoplasma Ab Qiagen Leipzig Germany. h = Se, sensitivity. i = Sp, specificity.

**Table 3B:** Characteristics of the six eligible studies on sheep included in a meta-analysis for estimating seroprevalence of *Toxoplasma gondii* in the Nordic-Baltic region.

Authors	Year of publication	Country	Study period	Sample type	Serological method	Se^e^	Sp^f^	Total no. of sheep ≤1 year of age (no. of seropositives)	Total no. of sheep >1 year of age (no. of seropositives)	Total no. of sheep of unknown age (no. of seropositives)	Apparent seroprevalence (%)
Skjerve et al., 1998	1998	Norway	1993–1993	Serum	ELISA^a^	n.r	>0.95	1940 (315)	0	0	16.2
Eglīte and Keidans, 2000	2000	Latvia	1993–1998	Serum	CF^b^	n.r	n.r	n.r	n.r	107 (6)	5.6
Eglīte and Keidans, 2000	2000	Latvia	1998–2000	Serum	LA^c^	n.r.	n.r.	n.r	n.r	20 (9)	45.0
Jokelainen et al., 2010	2010	Finland	2008–2008	Serum	DAT^d^	n.r.	n.r.	0	1940 (477)	0	24.6
Deksne et al., 2017	2017	Latvia	2012–2013	Serum	ELISA^a^	n.r.	n.r.	166 (18)	873 (161)	0	17.2
Tagel et al., 2019	2019	Estonia	2012–2013	Serum	DAT^d^	n.r	n.r	36 (4)	1511 (637)	52 (26)	41.7

n.r. = not reported. a = In-house Enzyme linked immunosorbent assay (ELISA). b = Unspecified commercial complement fixation test (CF). c = Latex agglutination test (LA). d = Commercial modified direct agglutination test (DAT), Toxo-Screen DA bioMérieux, Marcy-l'Étoile France [Cut-off, dilution of 1:40]. e = Se, sensitivity. f = Sp, specificity.

**Table 3C:** Characteristics of the three eligible studies on cattle included in the meta-analysis for estimating seroprevalence of *Toxoplasma gondii* in the Nordic-Baltic region.

Authors	Year of publication	Country	Study period	Sample type	Serological method	Se^d^	Sp^e^	Total no. of cattle ≤1 year of age (no. of seropositives)	Total no. of cattle >1 year of age (no. of seropositives)	Total no. of cattle of unknown age (no. of seropositives)	Apparent seroprevalence (%)
Eglīte and Keidans, 2000	2000	Latvia	1993–1998	Serum	CF^a^	n.r	n.r	n.r	n.r	254 (2)	0.8
Allén, 2016	2016	Finland	2013–2014	Meat juice	ELISA^b^	n.r	n.r	0	185 (15)	15 (0)	7.5
Jokelainen et al., 2017	2017	Estonia	2012–2013	Serum	DAT^c^	n.r.	n.r.	n.r	3679 (707)	312 (36)	18.6

n.r. = not reported. a = Unspecified commercial complement fixation test (CF). b = Commercial ELISA, ID Screen Toxoplasmosis Indirect Multi-species IDvet Innovative Diagnostics Montpellier France) [Cut-off, S/P = 50%]. c = Commercial modified direct agglutination test (DAT), Toxo-Screen DA bioMérieux, Marcy-l'Étoile France [Cut-off, dilution of 1:100]. d = Se, sensitivity. e = Sp, specificity.

**Table 3D:** Characteristics of the six eligible studies on wild boars included in the meta-analysis for estimating seroprevalence of *Toxoplasma gondii* in the Nordic-Baltic region.

Authors	Year of publication	Country	Study period	Sample type	Serological method	Se^d^	Sp^e^	Total no. of wild boars ≤1 year of age (no. of seropositives)	Total no. of wild boars >1 year of age (no. of seropositives)	Total no. of wild boars of unknown age (no. of seropositives)	Apparent seroprevalence (%)
Jokelainen et al., 2012	2012	Finland	2007–2008	Serum	DAT^a^	n.r.	n.r.	24 (7)	166 (54)	7 (4)	33.0
Deksne and Kirjušina, 2013	2013	Latvia	2010–2011	Meat juice	ELISA^b^	n.r.	n.r.	n.r.	n.r.	606 (201)	33.3
Jokelainen et al., 2015	2015	Estonia	2012–2013	Meat juice	DAT^a^	n.r.	n.r.	156 (35)	185 (51)	130 (27)	24.0
Wallander et al., 2015	2015	Sweden	2005–2011	Serum	ELISA^b^	0.79	0.85	275 (94)	205 (113)	847 (450)	49.5
Malmsten et al., 2018	2018	Sweden	2013–2015	Serum	ELISA^b^	n.r.	n.r.	n.r.	n.r.	276 (80)	29.0
Laforet et al., 2019	2019	Denmark	2016–2018	Serum	ELISA^c^, DAT^a^	n.r.	n.r.	38 (6)	61 (24)	2 (0)	29.7

n.r. = not reported. a = Commercial modified direct agglutination test (DAT), Toxo-Screen DA bioMérieux, Marcy-l'Étoile France [Cut-off, dilution of 1:40]. b = In-house Enzyme linked immunosorbent assay (ELISA). c = Commercial ELISA, ID Screen Toxoplasmosis Indirect Multi-species IDvet Innovative Diagnostics Montpellier France) [Cut-off, S/P = 50%]. d = Se, sensitivity. e = Sp, specificity.

**Table 3E:** Characteristics of the four eligible studies on moose included in the meta-analysis for estimating seroprevalence of *Toxoplasma gondii* in the Nordic-Baltic region.

Authors	Year of publication	Country	Study period	Sample type	Serological method	Se^b^	Sp^c^	Total no. of moose ≤1 year of age (no. of seropositives)	Total no. of moose >1 year of age (no. of seropositives)	Total no. of moose of unknown age (no. of seropositives)	Apparent seroprevalence (%)
Vikøren et al., 2004	2004	Norway	1992–2000	Serum	DAT^a^	n.r.	n.r.	607 (27)	1468 (233)	67 (10)	12.6
Jokelainen et al., 2010	2010	Finland	2008–2009	Serum	DAT^a^	n.r.	n.r.	454 (24)	729 (90)	32 (2)	9.5
Malmsten et al., 2011	2011	Sweden	2000–2005	Serum	DAT^a^	n.r.	n.r.	122 (17)	295 (68)	0	20.4
Remes et al., 2018	2018	Estonia	2015–2015	Serum or plasma	DAT^a^	n.r.	n.r.	143 (18)	316 (91)	4 (2)	24.0

a = Commercial modified direct agglutination test (DAT), Toxo-Screen DA bioMérieux, Marcy-l'Étoile France [Cut-off, dilution of 1:40]. n.r. = not reported. b = Se, sensitivity. c = Sp, specificity.

Different serological methods were used in the studies (Table 3A, Table 3B, Table 3C, Table 3D, Table 3E). For example, an enzyme linked immunosorbent assay (ELISA, in-house or commercial) was used in most studies on domestic pigs (n = 10), whereas a modified direct agglutination test (DAT, commercial) was used in all studies on moose (n = 4). Eight studies reported both the sensitivity and the specificity of the serological test used; seven of these studies were on domestic pigs.

### 3.2 Heterogeneity in the seroprevalence estimates and subgroup analyses

**Fig. 3:**
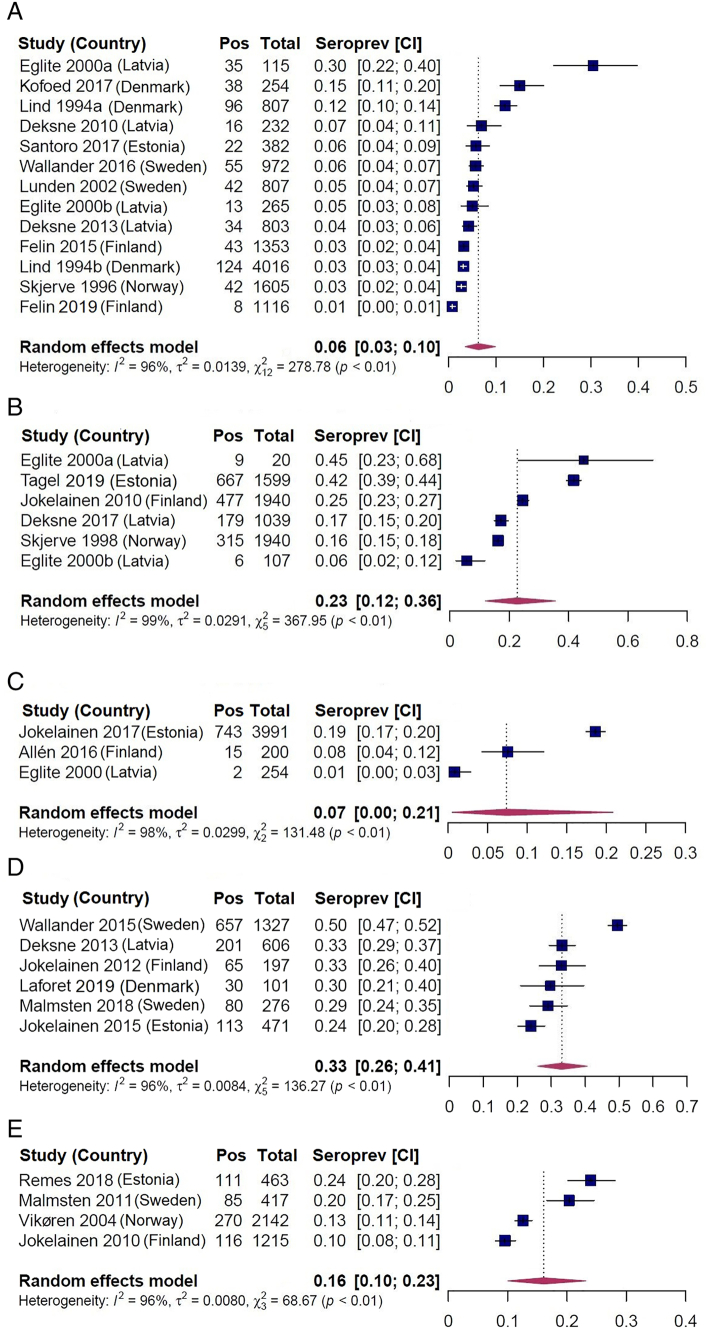
A. Forest plot of *Toxoplasma gondii* seroprevalence in domestic pigs (*Sus scrofa domesticus*) in the Nordic-Baltic region estimated with random-effects model ignoring the effect of geography, production system, and age. References: (Lind et al., 1994; Skjerve et al., 1996; Eglīte and Keidans, 2000; Lundén et al., 2002; Deksne, 2010; Deksne and Kirjušina, 2013; Felin et al., 2015; Wallander et al., 2016; Kofoed et al., 2017; Santoro et al., 2017; Felin et al., 2019). B. Forest plot of *Toxoplasma gondii* seroprevalence in sheep (*Ovis aries*) in the Nordic-Baltic region with random-effects model ignoring the effect of geography and age. References: (Skjerve et al., 1998; Eglīte and Keidans, 2000; Jokelainen et al., 2010; Deksne et al., 2017; Tagel et al., 2019). C. Forest plot of *Toxoplasma gondii* seroprevalence in cattle (*Bos taurus*) in the Nordic-Baltic region with random-effects model ignoring the effect of geography and age. References: (Eglīte and Keidans, 2000; Allén, 2016; Jokelainen et al., 2017). D. Forest plot of *Toxoplasma gondii* seroprevalence in wild boars (*Sus scrofa*) in the Nordic-Baltic region with random-effects model ignoring the effect of geography and age. References: (Jokelainen et al., 2012; Deksne and Kirjušina, 2013; Jokelainen et al., 2015; Wallander et al., 2015; Malmsten et al., 2018; Laforet et al., 2019). E. Forest plot of *Toxoplasma gondii* seroprevalence in moose (*Alces alces*) in the Nordic-Baltic region with random-effects model ignoring the effect of geography and age. References: (Vikøren et al., 2004; Jokelainen et al., 2010; Malmsten et al., 2011; Remes et al., 2018). Pos = number of animals that tested seropositive. Total = number of animals sampled. Seroprev [CI] = estimated apparent seroprevalence and its 95% confidence interval (CI), presented in descending order.

The *T. gondii* seroprevalence estimates varied markedly among the included studies: the crude range was 1–30% in domestic pigs, 6–45% in sheep, 1–19% in cattle, 24–50% in wild boars, and 10–24% in moose. For all host species, there was significant heterogeneity among the seroprevalence estimates from the included studies (Fig. 3A to E, Table 4). The highest pooled seroprevalence estimate (PSP) was observed for wild boars (PSP = 33%, CI_95%_: 26–41%) followed by sheep (PSP = 23%, CI_95%_: 12–36%), moose (PSP = 16%, CI_95%_: 10–23%), cattle (PSP = 7%, CI_95%_: 1–21%), and domestic pigs (PSP = 6%, CI_95%_: 3–10%). For all host species, the results from the leave-one-out analyses showed that the pooled seroprevalence estimates were not significantly influenced by any of the included studies (Appendix B, Figs. B.1 to B.5).

**Table 4:** Pooled *Toxoplasma gondii* seroprevalence estimates for five host species in the Nordic-Baltic region, 2018.

Host species	No. of studies	No. seropositive animals	Total no. of animals	Pooled seroprevalence (%) (95% CI)	Heterogeneity		
					Q	Q-P	I^2^%
Domestic pig	13	568	12,727	6.3 (3.5–10.0)	278.8	<0.001	98.0
Sheep	6	1653	6645	22.7 (12.0–36.0)	367.9	<0.001	99.1
Cattle	3	760	4445	7.4 (1.0–21.0)	131.5	<0.001	98.0
Wild boar	6	1146	2978	33.1 (26.0–41.0)	136.3	<0.001	93.5
Moose	4	582	4237	16.1 (10.0–23.2)	68.7	<0.001	96.7

I^2^ = Inverse variance index; Q = Cochran's Q test for heterogeneity; Q-P = probability value of Cochran's Q test for heterogeneity.

In all host species except for wild boars, the pooled seroprevalence estimate of *T. gondii* was significantly higher in old than in young animals (Table 5). The lowest pooled seroprevalence estimates in both the age groups were recorded in domestic pigs (PSP_young_ = 4%, CI_95%_: 2–6% and PSP_old_ = 18%, CI_95%_: 12–25%, respectively), and the highest were recorded in wild boars (PSP_young_ = 26%, CI_95%_: 16–37% and PSP_old_ = 38%, CI_95%_: 28–49%, respectively). Heterogeneity in the seroprevalence estimates between studies in the two age groups varied from moderate (young sheep, I^2^ = 49% and young wild boars, I^2^ = 71%) to high (young pigs, I^2^ = 90%; old pigs, I^2^ = 91%; old sheep, I^2^ = 99%; old wild boars, I^2^ = 91%; young moose, I^2^ = 85%, and old moose, I^2^ = 94%).

**Table 5:** Summary of estimated pooled *Toxoplasma gondii* seroprevalence and heterogeneity measures by age groups (young ≤1 year, old >1 year) in domestic pigs, sheep, wild boars and moose using mixed effects model as part of a systematic review for the Nordic and Baltic countries.

Host species	Age group	No. of positive animals	Total no. of animals	Pooled seroprevalence (%) (95% CI)	Heterogeneity			Statistical effect of age	Study [reference]
					Q	Q-P	I^2^ (%)	P-value	
Domestic pig^a^	Young	181	5048	4.0 (2.0–6.3)	69.6	<0.01	90.0	<0.0001	Lind et al., 1994; Lundén et al., 2002; Deksne, 2010; Felin et al., 2015; Wallander et al., 2016; Kofoed et al., 2017; Santoro et al., 2017; Felin et al., 2019
	Old	138	802	18.1 (12.0–25.2)	32.8	<0.01	91.0		Lind et al., 1994; Lundén et al., 2002; Kofoed et al., 2017; Santoro et al., 2017
Sheep^b^	Young	337	2142	13.1 (5.6–23.0)	3.9	0.14	49.0	0.04	Skjerve et al., 1998; Deksne et al., 2017; Tagel et al., 2019
	Old	1275	4324	27.8 (17.9–38.9)	188.4	<0.01	99.0		Jokelainen et al., 2010; Deksne et al., 2017; Tagel et al., 2019
Wild boar^c^	Young	142	493	25.7 (16.0–36.7)	10.2	0.02	71.0	0.10	Jokelainen et al., 2012; Jokelainen et al., 2015; Wallander et al., 2015; Laforet et al., 2019
	Old	242	617	38.4 (28.0–49.4)	35.3	<0.01	91.0		Jokelainen et al., 2012; Jokelainen et al., 2015; Wallander et al., 2015; Laforet et al., 2019
Moose^d^	Young	86	1326	8.3 (4.0–14.0)	46.3	<0.001	85.0	0.01	Vikøren et al., 2004; Jokelainen et al., 2010; Malmsten et al., 2011; Remes et al., 2018
	Old	482	2808	19.4 (13.0–26.7)	20.5	<0.001	94.0		Vikøren et al., 2004; Jokelainen et al., 2010; Malmsten et al., 2011; Remes et al., 2018

I^2^ = Inverse variance index; Q = Cochran's Q test for heterogeneity; Q-P = probability value of Cochran's Q test for heterogeneity. a = Data extracted from eight studies as five studies did not report age in domestic pigs. In total, the age group of 6877 out of 12,727 animals was not reported. b = Data extracted from four studies as two studies did not report age in sheep. In total, the age group of 179 out of 6645 animals was not reported. c = Data extracted from four studies as two studies did not report age in wild boars. In total, the age group of 1868 out of 2978 animals not reported. d = Data extracted from all four studies reporting age group of the moose. In total, the age of 103 out of 4237 animals was not reported.

The pooled seroprevalence estimate was 3% (CI_95%_: 0.3–7%, I^2^ = 96%) in indoor pigs and 8% (CI_95%_: 2–16, I^2^ = 98%) in outdoor pigs. The difference between the pooled seroprevalence estimates in domestic pigs from these two production systems was not statistically significant (P = 0.11).

## 4 Discussion

This systematic literature review and meta-analysis estimated *T. gondii* seroprevalence in domestic pigs, sheep, cattle, wild boars, and moose in the Nordic-Baltic region, and identified important data gaps. The results of this study summarize the extent of reported exposure to *T. gondii* in different animal species in the Nordic-Baltic region, which is of importance in the context of food safety. Particularly from the Baltic states, limited data have been previously available, whereas several studies have been conducted since the year 2013 and were included in this systematic review.

The host species covered in this study included both farm animals and game animals. Domestic pigs and wild boars are omnivores and may acquire *T. gondii* infection by eating sporulated oocysts, shed in unsporulated form by infected felids, or by eating tissues of infected animals. Sheep, cattle, and moose as herbivores likely acquire the infection from sporulated oocysts. All included studies reported seropositive animals, indicating exposure to *T. gondii* is widespread in the region.

A wide range of *T. gondii* seroprevalence estimates was observed across the different studies. Because of the high level of heterogeneity between the studies, the pooled seroprevalence estimates presented in this study should be interpreted together with the 95% CIs.

Among the farm animals, domestic pigs had the lowest pooled seroprevalence estimate (PSP = 6%, CI_95%_: 3–10%), which was on the lower end of the wide range for seroprevalence estimates for pigs from Europe (0.4–64%) (Dubey, 2009b). The highest pooled seroprevalence estimate among farm animals was observed in sheep (PSP = 23%, CI_95%_: 12–36%) and the estimate was close to the mid-range of the seroprevalence estimates for sheep reported in Europe (4–66%) (Dubey, 2009a). From an interpretative perspective, the pooled seroprevalence estimate in cattle (PSP = 7%, CI_95%_: 1–21%) may be of limited importance due to the low number of studies and high heterogeneity in seroprevalence between them. The relevance of serology for screening of *T. gondii* in cattle is debatable due to reports of poor correlation between seropositivity and direct detection of the parasites (Opsteegh et al., 2011; Opsteegh et al., 2016).

Among the host species included in our study, wild boars had the highest pooled seroprevalence estimate (PSP = 33%, CI_95%_: 26–41%), which was in the mid-range of the seroprevalence estimates for wild boars in Europe (5–57%) (Rostami et al., 2017). *Toxoplasma gondii* is common in wild boar populations throughout the world; the pooled seroprevalence in Europe has been estimated to be 26% (CI: 21–30%) (Rostami et al., 2017). For moose, the pooled seroprevalence estimate in this study (PSP = 16%, CI_95%_: 10–23%) was also in line with the seroprevalence estimates from other parts of the world (USA 10%; Canada 15%) (Siepierski et al., 1990; Verma et al., 2016).

A higher seroprevalence was observed in animals >1 year of age than in younger animals in all host species covered in our study except wild boars, and similar results have been reported in other studies (Opsteegh et al., 2016). In general, higher seroprevalence in older animals likely reflects a longer period of exposure, which increases the probability of acquiring the infection.

Dutch studies have reported higher seroprevalences (5% and 6%) in pigs from outdoor farms compared with pigs from intensive indoor farms (0 and 0.4%) (Kijlstra et al., 2004; van der Giessen et al., 2007). We did not find a statistically significant difference in the pooled seroprevalence estimates between pigs from indoor (PSP = 3%; CI_95%_: 0.3–7%, I^2^ = 96%) and outdoor production systems (PSP = 8%; CI_95%_: 2–16, I^2^ = 98%) in our study, possibly because of large variation between the limited number of studies. The pooled seroprevalence estimate for pigs from outdoor farms in our study was higher than the estimates from the two Dutch studies.

The studies included in the meta-analysis used different serological methods, sample material, and cut-offs for seropositivity. The performance of serological tests varies between the type of samples (Hill et al., 2006) and the selected cut-offs (Felin et al., 2017). When the serological test is imperfect (sensitivity <100%; specificity <100%), the prevalence estimate obtained from the test is biased; however, this bias can be corrected if the sensitivity and specificity of the tests are known (Diggle, 2011). There is no ‘reference standard’ serological test with 100% sensitivity and 100% specificity for anti-*T. gondii* antibodies; therefore, it may be useful to apply a latent class approach to estimate the accuracy of a test (Gardner et al., 2010). In this study, we decided to work on apparent seroprevalence estimates from all the studies, without pooling the studies based on the serological test type, due to the lack of data on sensitivity and specificity of the tests needed for adjustment of the seroprevalence estimate. Hence, it is likely that the differences in the serological methods may have contributed to the heterogeneity seen in seroprevalence between the studies.

However, five studies on domestic pigs (Table 3A: (Lind et al., 1994; Lundén et al., 2002; Felin et al., 2015; Kofoed et al., 2017; Santoro et al., 2017)), two studies on sheep (Table 3B: (Deksne et al., 2017; Tagel et al., 2019)), three studies on wild boars (Table 3D: (Jokelainen et al., 2015; Wallander et al., 2015; Laforet et al., 2019)), and four studies on moose (Table 3E: (Vikøren et al., 2004; Jokelainen et al., 2010; Malmsten et al., 2011; Remes et al., 2018)) tested young and old animals using the same test and the same cut-off, and found the seroprevalence to be higher in old animals than in young animals as confirmed by the subgroup analysis (Table 5). Hence, the observed difference in seroprevalence by age group appears to be a direct effect of age and cannot be simply attributed to the choice of diagnostic test.

In meta-analysis, it is recommended to quantify and adjust for publication bias using statistical methods (Møller and Jennions, 2001). This is done to correct for missing studies to avoid overestimation of the true effect size. However, currently available methods such as Egger's regression test and the trim-and-fill method are not considered useful in studies on proportions (Murad et al., 2018). Additionally, the statistical power of the tests is affected by the presence of high heterogeneity and the limited number of studies (Lau et al., 2006). Therefore, we decided not to look for evidence of publication bias in our study.

Based on the included studies, a substantial proportion of animals investigated were seropositive, which indicates that animals raised or hunted for human consumption in the Nordic-Baltic region were commonly exposed to *T. gondii*. There was a large variation in seroprevalence estimates between the studies in the region. If the observed variations in animal seroprevalence estimates between the studies in the region represent spatial variations in prevalence, then this might partly explain the geographical variation in the reported seroprevalence in human estimates in this region (Lappalainen et al., 1992; Lebech et al., 1994; Pehk, 1994; Birgisdóttir et al., 2006; Findal et al., 2015; Lassen et al., 2016; Suvisaari et al., 2017; Siponen et al., 2019). However, the number of studies available was too low to identify spatial patterns at the country-level, and other differences, such as the age of the tested animals and the serological tests applied may also have affected the estimates. To clarify the sources of heterogeneity, more studies and more data on risk factors that are relevant to each host species are needed. Furthermore, it is important for future studies to report the age of the animals tested, mention the type of production system the animals raised for food are reared in and report the sensitivity, specificity, and cut-off of the serological test used, whenever possible.

## Appendix A Supplementary data

Appendix A Search strings used for identifying studies and the list of corresponding authors contacted to identify further studies. Image 1

Appendix B Leave one out analysis. Image 2

Appendix C Excel file containing all the data (studies identified, eligible studies, extracted data, and excluded studies). Image 3

Appendix D Excel file used for the data extraction. Image 4

Appendix E Instructions to the authors for the data-extraction sheet. Image 5
