# Supplementary material for: Seroprevalence of Toxoplasma gondii in domestic pigs, sheep, cattle, wild boars, and moose in the Nordic-Baltic region: A systematic review and meta-analysis
Source: Parasite Epidemiol Control. 2019 Mar 4;5:e00100. doi: 10.1016/j.parepi.2019.e00100 (PMC6411595; doi:10.1016/j.parepi.2019.e00100)
Supplement: Appendix A — Search strings used for identifying studies and the list of corresponding authors contacted to identify further studies. [file mmc1.pdf]

Appendix A

Table A.1. Search strings of authors AO and RB for the peer-reviewed articles on *Toxoplasma gondii* seroprevalence in domestic pigs, sheep, cattle, moose and wild boars on CAB Abstracts (1973 to 2018 Week 40) and OVID MEDLINE (1946-2018) databases from Nordic-Baltic countries

Species	Search string (AO)	Search string (RB)
Domestic pigs	1. Toxoplasma gondii.mp. [mp=abstract, title, original title, broad terms, heading words, identifiers, cabicodes]<br>2. limit 1 to yr="1990 -Current"<br>3. (prevalence or survey or proportion or positive* or risk factor* or seroprevalence).mp. [mp=abstract, title, original title, broad terms, heading words, identifiers, cabicodes]<br>4. limit 3 to yr="1990 -Current"<br>5. (Swine or pig* or boar* or piglet* or "Sus domesticus" or hog* or porcine or pork).mp. [mp=abstract, title, original title, broad terms, heading words, identifiers, cabicodes]<br>6. limit 5 to yr="1990 -Current"<br>7. (Denmark or Sweden or Norway or Finland or Iceland or Faroe Islands or Latvia or Lithuania or Estonia or Baltic* or Nordic* or Scandinavia* or Greenland).mp. [mp=abstract, title, original title, broad terms, heading words, identifiers, cabicodes]<br>8. limit 7 to yr="1990 -Current"<br>9. 2 and 4<br>10. 6 and 9<br>11. 8 and 10	1. Toxoplasma gondii.mp. [mp=abstract, title, original title, broad terms, heading words, identifiers, cabicodes]<br>2. (Prevalence or survey or study).mp. [mp=abstract, title, original title, broad terms, heading words, identifiers, cabicodes]<br>3. (Denmark or Danish or Sweden or swedish or Finland or finish or Iceland* or " Faroe Islands" or Greenland* or Norway or norwegian or Estonia* or Latvia* or Lithuania*).mp. [mp=abstract, title, original title, broad terms, heading words, identifiers, cabicodes]<br>4. 1 and 2 and 3<br>5. (Pig* or swine or sow* or "Sus scrofa domesticus" or piglet* or Barrow or boar or gilt* or farrow or shoat).mp. [mp=abstract, title, original title, broad terms, heading words, identifiers, cabicodes]<br>6. 4 and 5
Sheep	1. Toxoplasma gondii.mp. [mp=abstract, title, original title, broad terms, heading words, identifiers, cabicodes]<br>2. limit 1 to yr="1990 -Current"<br>3. (prevalence or survey or occurrence, proportion, positive, risk factor*, seroprevalence).mp. [mp=abstract, title, original title, broad terms, heading words, identifiers, cabicodes]<br>4. limit 3 to yr="1990 -Current"<br>5. (sheep or lamb* or ram* or ewe* or lamb* or ovine* or mutton).mp. [mp=abstract, title, original title, broad terms, heading words, identifiers, cabicodes]<br>6. limit 5 to yr="1990 -Current"<br>7. (Denmark or Sweden or Norway or Finland or Iceland or Faroe Islands or Latvia or Lithuania or Estonia or Baltic* or Nordic* or Scandinavia* or Greenland).mp.	1. Toxoplasma gondii.mp. [mp=abstract, title, original title, broad terms, heading words, identifiers, cabicodes]<br>2. (Prevalence or survey or study).mp. [mp=abstract, title, original title, broad terms, heading words, identifiers, cabicodes]<br>3. 1 and 2<br>4. (sheep or lamb* or "Ovis aries").mp. [mp=abstract, title, original title, broad terms, heading words, identifiers, cabicodes]<br>5. 3 and 4<br>6. (Denmark or Danish or Sweden or swedish or Finland or finish or Iceland* or " Faroe Islands" or Greenland* or Norway or norwegian or Estonia* or Latvia* or Lithuania*).mp. [mp=abstract, title, original title, broad terms, heading words, identifiers, cabicodes]

	[mp=abstract, title, original title, broad terms, heading words, identifiers, cabicodes]<br>8. limit 7 to yr="1990 -Current"<br>9. 2 and 4<br>10. 6 and 9<br>11. 8 and 10	7. 5 and 6
Cattle	1. Toxoplasma gondii.mp. [mp=abstract, title, original title, broad terms, heading words, identifiers, cabicodes]<br>2. limit 1 to yr="1990 -Current"<br>3. (prevalence or survey or proportion or positive* or risk factor* or seroprevalence).mp. [mp=abstract, title, original title, broad terms, heading words, identifiers, cabicodes]<br>4. limit 3 to yr="1990 -Current"<br>5. (cattle or bovine* or calf or beef or calves or cow or "Bos taurus").mp. [mp=abstract, title, original title, broad terms, heading words, identifiers, cabicodes]<br>6. limit 5 to yr="1990 -Current"<br>7. (Denmark or Sweden or Norway or Finland or Iceland or Faroe Islands or Latvia or Lithuania or Estonia or Baltic* or Nordic* or Scandinavia* or Greenland).mp. [mp=abstract, title, original title, broad terms, heading words, identifiers, cabicodes]<br>8. limit 7 to yr="1990 -Current"<br>9. 1 and 3<br>10. 6 and 9<br>11. 8 and 10	1. Toxoplasma gondii.mp. [mp=abstract, title, original title, broad terms, heading words, identifiers, cabicodes]<br>2. (Prevalence or survey or study or presence or insidence).mp. [mp=abstract, title, original title, broad terms, heading words, identifiers, cabicodes]<br>3. 1 and 2<br>4. (Cattle or cow* or bull* or calf or calves or oxen or "Bos taurus").mp. [mp=abstract, title, original title, broad terms, heading words, identifiers, cabicodes]<br>5. 3 and 4<br>6. (Denmark or Danish or Sweden or swedish or Finland or finish or Iceland* or " Faroe Islands" or Greenland* or Norway or norwegian or Estonia* or Latvia* or Lithuania*).mp. [mp=abstract, title, original title, broad terms, heading words, identifiers, cabicodes]<br>7. 5 and 6
Wild boars	1. Toxoplasma gondii.mp. [mp=abstract, title, original title, broad terms, heading words, identifiers, cabicodes]<br>2. limit 1 to yr="1990 -Current"<br>3. (prevalence or survey or proportion or positive* or risk factor* or seroprevalence).mp. [mp=abstract, title, original title, broad terms, heading words, identifiers, cabicodes]<br>4. limit 3 to yr="1990 -Current"<br>5. (wild boar or wild swine or wild pig* or wild pork).mp. [mp=abstract, title, original title, broad terms, heading words, identifiers, cabicodes]<br>6. limit 5 to yr="1990 -Current"<br>7. (Denmark or Sweden or Norway or Finland or Iceland or Faroe Islands or Latvia or Lithuania or Estonia or Baltic* or Nordic* or Scandinavia* or Greenland).mp. [mp=abstract, title, original title, broad terms, heading words, identifiers, cabicodes]	1. Toxoplasma gondii.mp. [mp=abstract, title, original title, broad terms, heading words, identifiers, cabicodes]<br>2. (Prevalence or survey or study).mp. [mp=abstract, title, original title, broad terms, heading words, identifiers, cabicodes]<br>3. (Denmark or Danish or Sweden or swedish or Finland or finish or Iceland* or " Faroe Islands" or Greenland* or Norway or norwegian or Estonia* or Latvia* or Lithuania*).mp. [mp=abstract, title, original title, broad terms, heading words, identifiers, cabicodes]<br>4. 1 and 2 and 3<br>5. ("wild boar*" or "sus scrofa" or "wild swine" or "wild pig*").mp. [mp=abstract, title, original title, broad terms, heading words, identifiers, cabicodes]<br>6. 4 and 5

	8. limit 7 to yr="1990 -Current"<br>9. 2 and 4<br>10. 5 and 9<br>11. 7 and 10	
Moose	1. Toxoplasma gondii.mp. [mp=abstract, title, original title, broad terms, heading words, identifiers, cabicodes]<br>2. limit 1 to yr="1990 -Current"<br>3. (prevalence or survey or proportion or positive* or risk factor* or seroprevalence).mp. [mp=abstract, title, original title, broad terms, heading words, identifiers, cabicodes]<br>4. limit 3 to yr="1990 -Current"<br>5. (moose or "Alces alces" or elf or cervidae or cervid or capreolinae).mp. [mp=abstract, title, original title, broad terms, heading words, identifiers, cabicodes]<br>6. limit 5 to yr="1990 -Current"<br>7. (Denmark or Sweden or Norway or Finland or Iceland or Faroe Islands or Latvia or Lithuania or Estonia or Baltic* or Nordic* or Scandinavia* or Greenland).mp. [mp=abstract, title, original title, broad terms, heading words, identifiers, cabicodes]<br>8. limit 7 to yr="1990 -Current"<br>9. 2 and 4<br>10. 6 and 9<br>11. 8 and 10	1. Toxoplasma gondii.mp. [mp=abstract, title, original title, broad terms, heading words, identifiers, cabicodes]<br>2. (Prevalence or survey or study).mp. [mp=abstract, title, original title, broad terms, heading words, identifiers, cabicodes]<br>3. (Denmark or Danish or Sweden or swedish or Finland or finish or Iceland* or " Faroe Islands" or Greenland* or Norway or norwegian or Estonia* or Latvia* or Lithuania*).mp. [mp=abstract, title, original title, broad terms, heading words, identifiers, cabicodes]<br>4. 1 and 2 and 3<br>5. (moose or "alces alces" or "elk").mp. [mp=abstract, title, original title, broad terms, heading words, identifiers, cabicodes]<br>6. 4 and 5

Table A.2. Search string on ProQuest database for published theses on *Toxoplasma gondii* seroprevalence in domestic pigs, sheep, cattle, moose and wild boars on CAB Abstracts (1973 to 2018 Week 40) and OVID MEDLINE (1946-2018) databases from Nordic-Baltic countries

"Toxoplasma gondii" AND (schloc.exact("Sweden" OR "Denmark") AND subt.exact("parasitology" OR "pathology" OR "veterinary services" OR "public health" OR "animal diseases" OR "epidemiology" OR "biology" OR "animals" OR "animal sciences" OR "agriculture" OR "health education" OR "livestock") AND pd(19900101-20181231))

Table A.3. List of corresponding authors contacted to identify further studies on *Toxoplasma gondii* seroprevalence from the Nordic-Baltic region

Author	Country	Contact method	Response (Yes/No)	No. of further studies identified
Malmsten, A	Sweden	Email	No	0
Oksanen, A[a]	Finland	Email	Yes	1
Ljungström, B	Sweden	Email	Yes	0
Wallander, C	Sweden	Email	No	0
Eglīte, I	Latvia	Email	Yes	0
Felin, E	Finland	Email	Yes	1
Skjerve, E	Norway	Email	Yes	1
Deksne, G	Latvia	Email	Yes	1
Enemark, H	Norway	Email	Yes	0
Malmsten, J	Sweden	Email	No	0
Tagel, M	Estonia	Email	Yes	0
Lind, P	Denmark	Email, Phone	Yes	0
Jokelainen, P	Finland	Email	Yes	1
Hirvelä-Koski, V[b]	Finland	Email	Yes	0

a= Author referred by the corresponding author b
