# Supplementary material for: Seroprevalence of Toxoplasma gondii in domestic pigs, sheep, cattle, wild boars, and moose in the Nordic-Baltic region: A systematic review and meta-analysis
Source: Parasite Epidemiol Control. 2019 Mar 4;5:e00100. doi: 10.1016/j.parepi.2019.e00100 (PMC6411595; doi:10.1016/j.parepi.2019.e00100)
Supplement: Appendix B — Leave one out analysis. [file mmc2.pdf]

B Leave one out analysis on studies reporting seroprevalence of Toxoplasma gondii in domestic pigs, sheep, cattle, wild boars and moose in the Nordic-Baltic region.

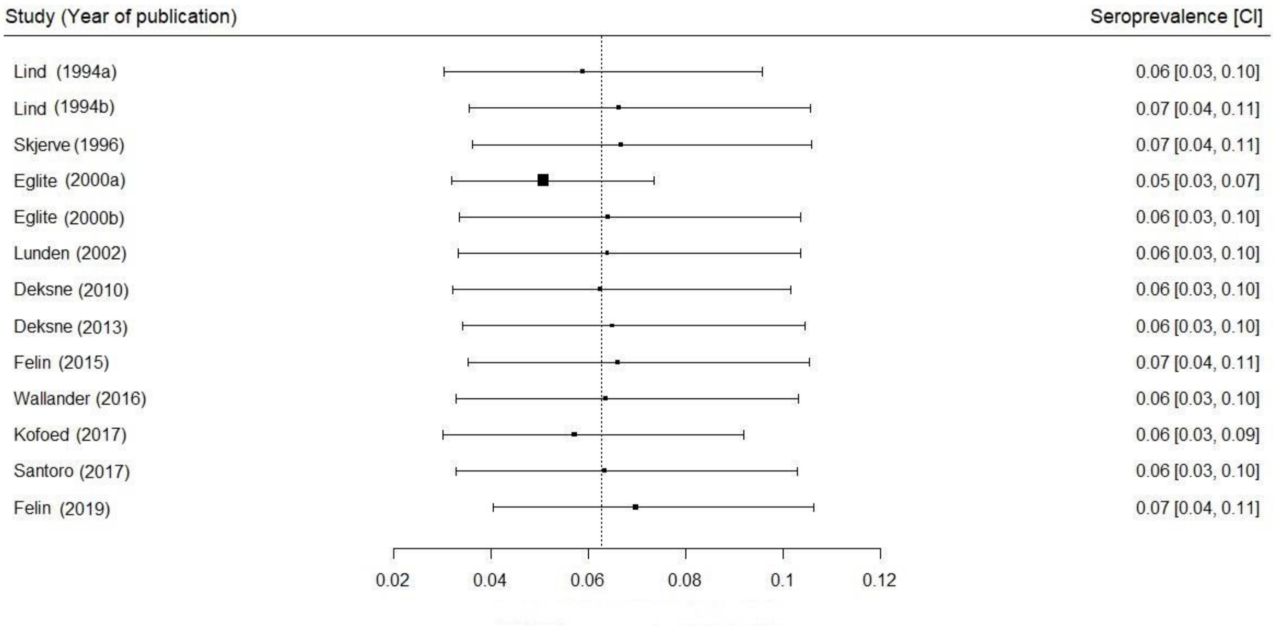

Figure B.1. Leave-one out analysis on the studies reporting seroprevalence of *Toxoplasma gondii* in domestic pigs. Overall pooled seroprevalence for all 13 studies was 0.06 indicated by the vertical dotted line ($CI_{95\%}$= 0.03–0.10, I^2= 98%). Each of the 13 pooled seroprevalence estimates calculated by leaving out each of the individual studies one by one were all well within the 95% confidence limits of the overall seroprevalence of all 13 studies.

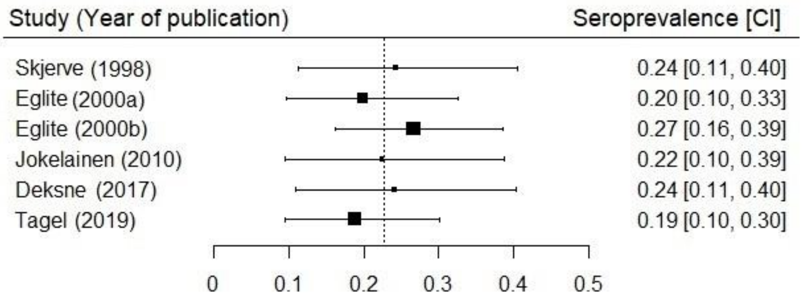

Figure B.2. Leave-one out analysis on the studies reporting seroprevalence of *Toxoplasma gondii* in sheep. Overall pooled seroprevalence for all six studies was 0.23 indicated by the vertical dotted line ($CI_{95\%}$= 0.12–0.36, I^2= 99%). Each of the six pooled seroprevalence estimates calculated by leaving out each of the individual studies one by one were all well within the 95% confidence limits of the overall seroprevalence of all six studies.

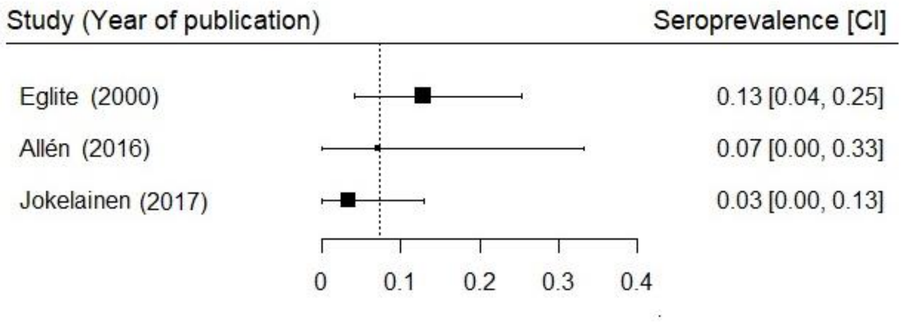

Figure B.3. Leave-one out analysis on the studies reporting seroprevalence of *Toxoplasma gondii* in cattle. Overall pooled seroprevalence for all three studies was 0.07 indicated by the vertical dotted line ($CI_{95\%}$= 0.01–0.21, I^2= 98%). Each of the three pooled seroprevalence estimates calculated by leaving out each of the individual studies one by one were all well within the 95% confidence limits of the overall seroprevalence of all three studies.

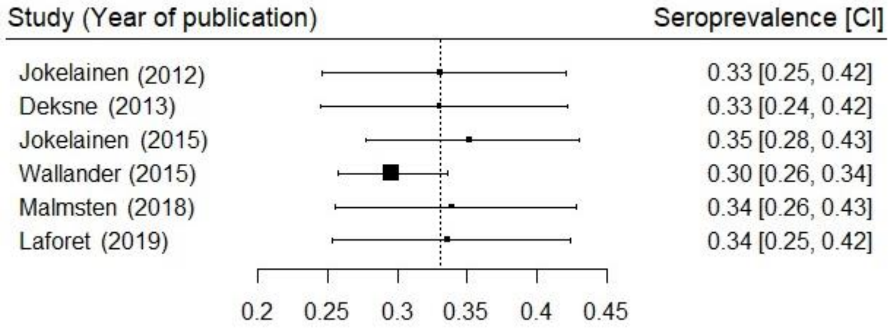

Figure B.4. Leave-one out analysis on the studies reporting seroprevalence of *Toxoplasma gondii* in wild boars. Overall pooled seroprevalence for all six studies was 0.33 indicated by the vertical dotted line ($CI_{95\%}$= 0.26–0.41, I^2= 94%). Each of the six pooled seroprevalence estimates calculated by leaving out each of the individual studies one by one were all well within the 95% confidence limits of the overall seroprevalence of all six studies.

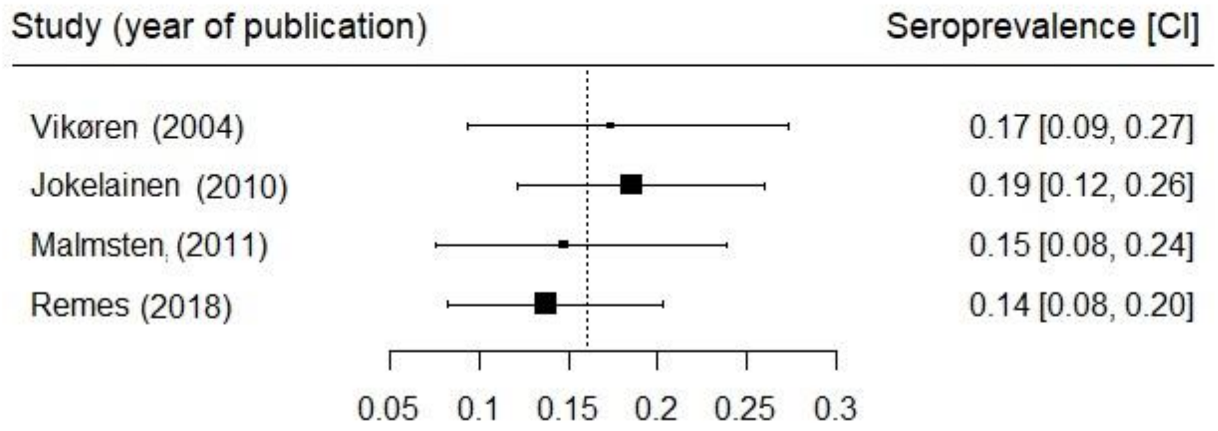

Figure B.5. Leave-one out analysis on the studies reporting seroprevalence of *Toxoplasma gondii* in moose. Overall pooled seroprevalence for all four studies was 0.16 indicated by the vertical dotted line ($CI_{95\%}$= 0.10–0.23, I^2= 99%). Each of the four pooled seroprevalence estimates calculated by leaving out each of the individual studies one by one were all well within the 95% confidence limits of the overall seroprevalence of all four studies.

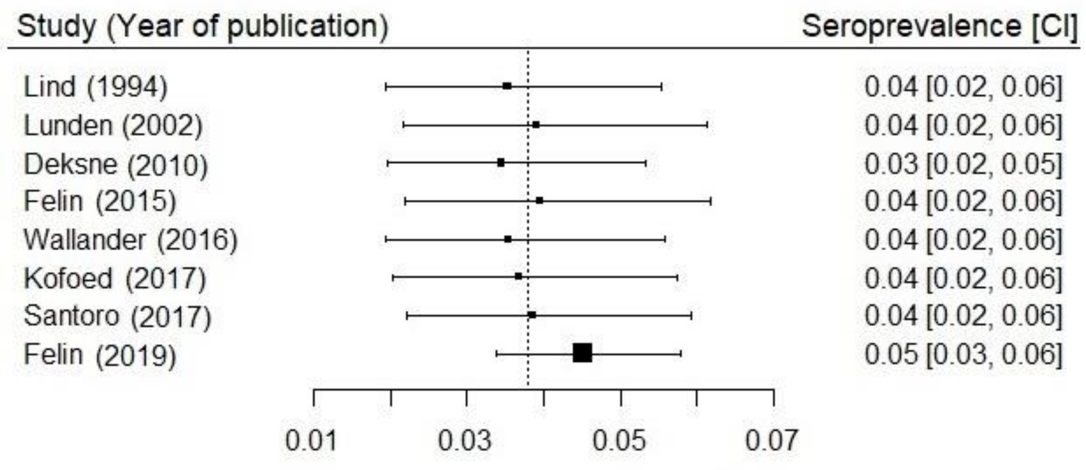

Figure B.6. Leave-one out analysis on studies reporting *Toxoplasma gondii* seroprevalence in (≤ 1 year = young) included in the young pig subgroup category. Overall pooled seroprevalence for eight studies was 4.0 % indicated by the vertical dotted line ($CI_{95\%}$: 2–6.3%, I^2= 90.0%). Each of the eight pooled seroprevalence estimates calculated by leaving out each of the individual studies one by one were all well within the 95% confidence limits of the overall seroprevalence of eight studies.

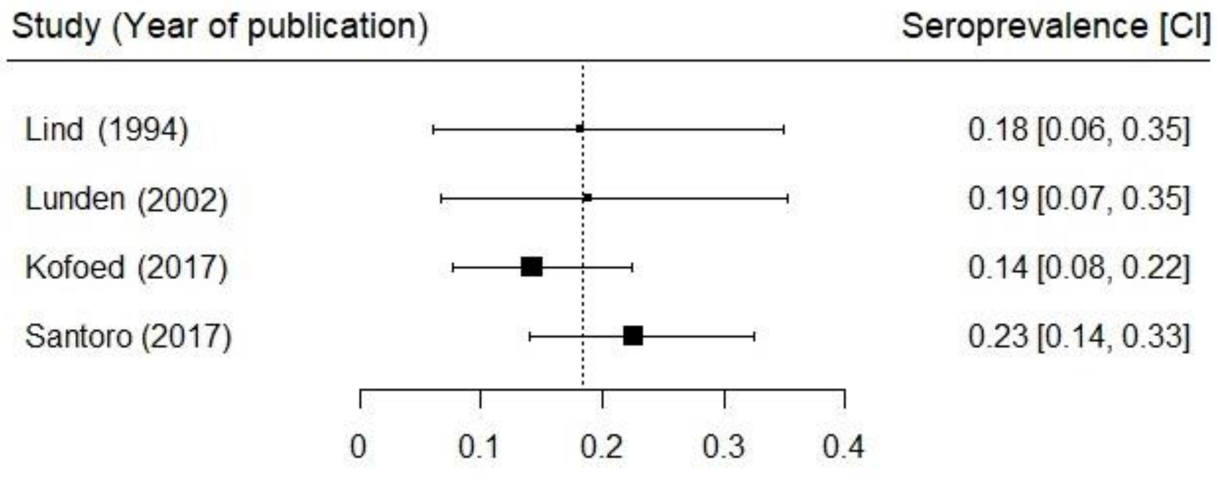

Figure B.7. Leave-one out analysis on studies reporting *Toxoplasma gondii* seroprevalence in old domestic pig studies (> 1 year = old) included in the old pig subgroup category. Overall pooled seroprevalence for four studies was 18.1% indicated by the vertical dotted line ($CI_{95\%}$: 12–25.2%, I^2= 91.0%). Each of the four pooled seroprevalence estimates calculated by leaving out each of the individual studies one by one were all well within the 95% confidence limits of the overall seroprevalence of four studies.

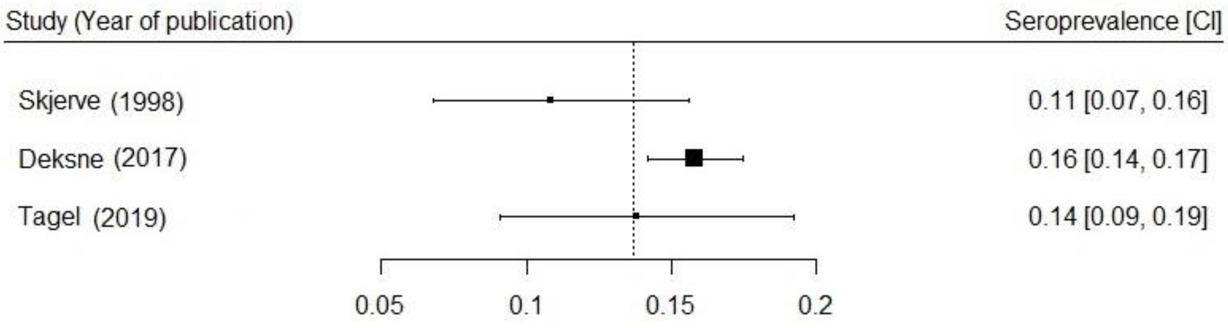

Figure B.8. Leave-one out analysis on studies reporting *Toxoplasma gondii* seroprevalence in young sheep studies (≤ 1 year = young) included in the young sheep subgroup. Overall pooled seroprevalence for three studies was 13.1% indicated by the vertical dotted line ($CI_{95\%}$: 5.6–23%, I^2= 49.0%). Each of the three pooled seroprevalence estimates calculated by leaving out each of the individual studies one by one were all well within the 95% confidence limits of the overall seroprevalence of three studies.

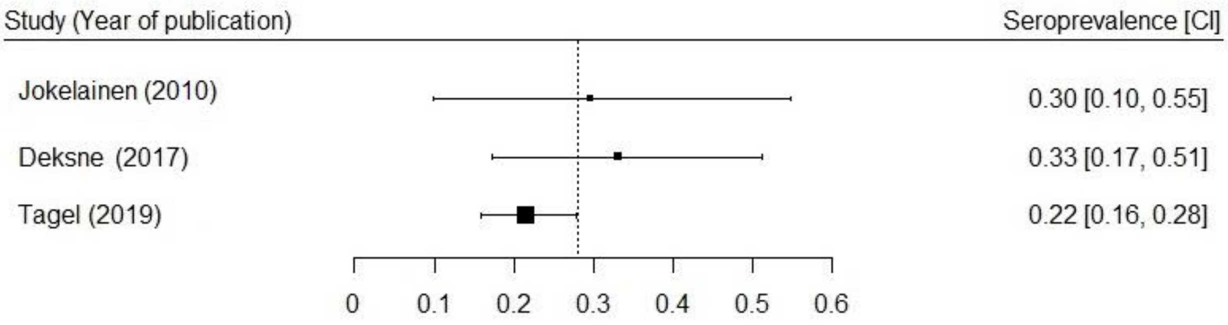

Figure B.9. Leave-one out analysis on studies reporting *Toxoplasma gondii* seroprevalence in old sheep (> 1 year = old) included in the old sheep subgroup. Overall pooled seroprevalence for three studies was 28.0% indicated by the vertical dotted line ($CI_{95\%}$: 18–39%, I^2= 99.0%). Each of the three pooled seroprevalence estimates calculated by leaving out each of the individual studies one by one were all well within the 95% confidence limits of the overall seroprevalence of three studies.

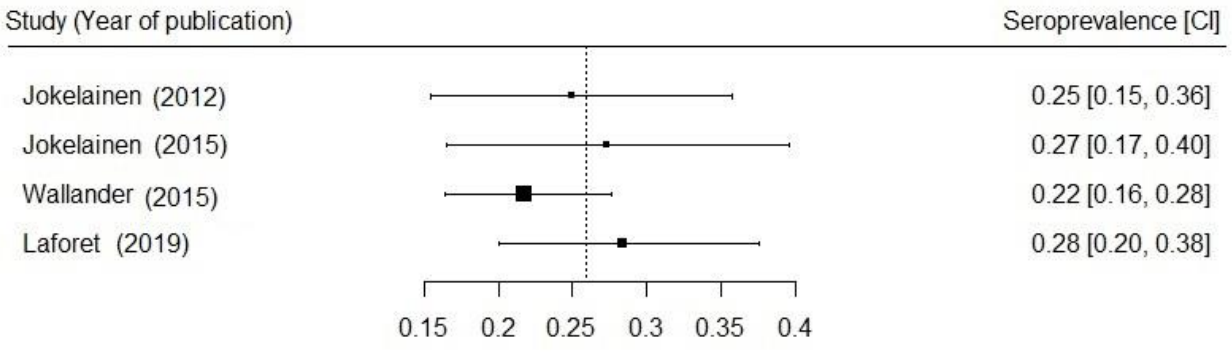

Figure B.10. Leave-one out analysis on studies reporting *Toxoplasma gondii* seroprevalence in young wild boars (≤ 1 year = young) included in the young wild boar subgroup. Overall pooled seroprevalence for three studies was 26.0% indicated by the vertical dotted line ($CI_{95\%}$: 16–37%, I^2= 71.0%). Each of the four pooled seroprevalence estimates calculated by leaving out each of the individual studies one by one were all well within the 95% confidence limits of the overall seroprevalence of four studies.

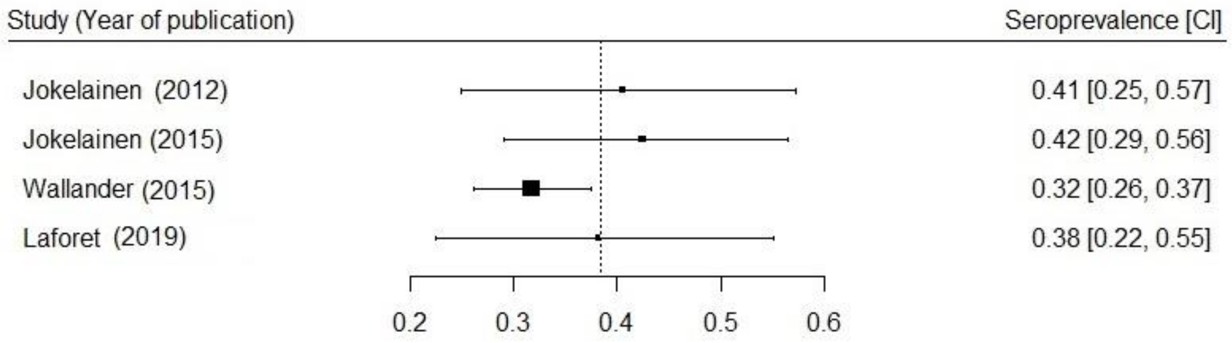

Figure B.11. Leave-one out analysis on studies reporting *Toxoplasma gondii* seroprevalence in old wild boars (> 1 year = old) included in the old wild boar subgroup. Overall pooled seroprevalence for three studies was 38.0% indicated by the vertical dotted line ($CI_{95\%}$: 28–49%, I^2= 91.0%). Each of the four pooled seroprevalence estimates calculated by leaving out each of the individual studies one by one were all well within the 95% confidence limits of the overall seroprevalence of four studies.

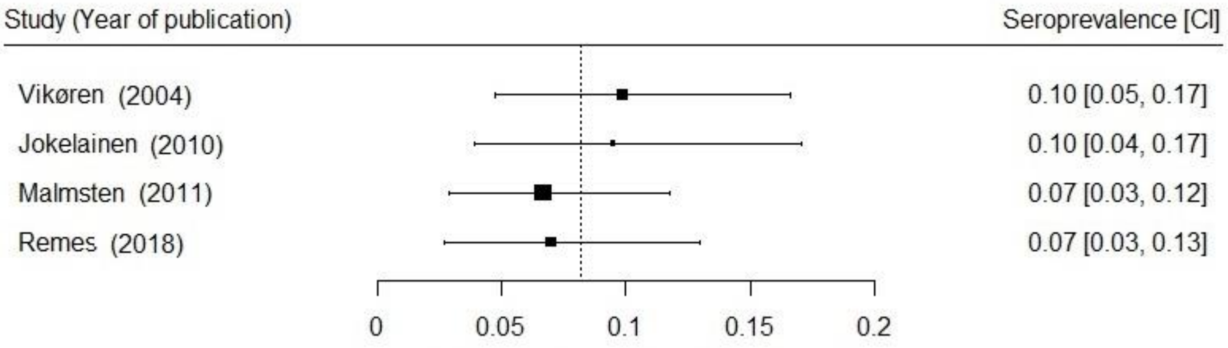

Figure B.12. Leave-one out analysis on studies reporting *Toxoplasma gondii* seroprevalence in young moose (≤ 1 year = young) included in the young moose subgroup. Overall pooled seroprevalence for three studies was 8.0% indicated by the vertical dotted line ($CI_{95\%}$: 4–14%, I^2= 85.0%). Each of the four pooled seroprevalence estimates calculated by leaving out each of the individual studies one by one were all well within the 95% confidence limits of the overall seroprevalence of four studies.

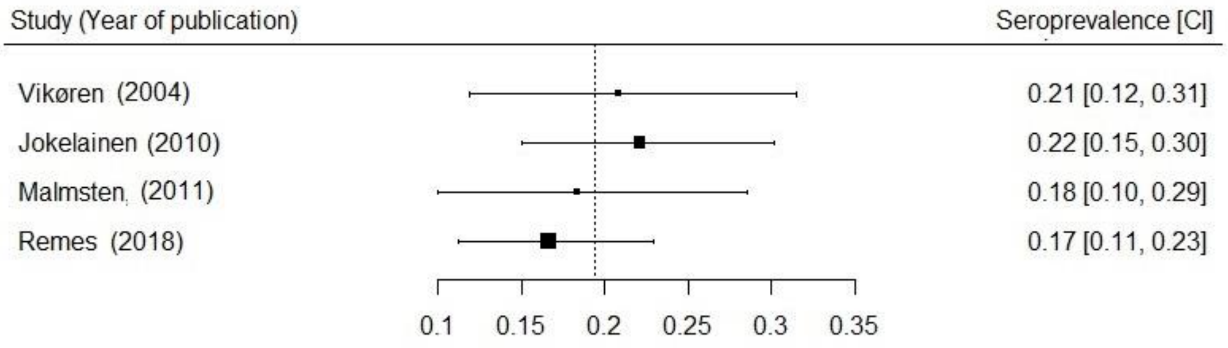

Figure B.13. Leave-one out analysis on studies reporting *Toxoplasma gondii* seroprevalence in old moose (> 1 year = old) included in the old moose subgroup. Overall pooled seroprevalence for four studies was 19 % indicated by the vertical dotted line ($CI_{95\%}$: 13–27%, I^2= 94.0%). Each of the four pooled seroprevalence estimate calculated by leaving out each of the individual studies one by one were all well within the 95% confidence limits of the overall seroprevalence of four studies.

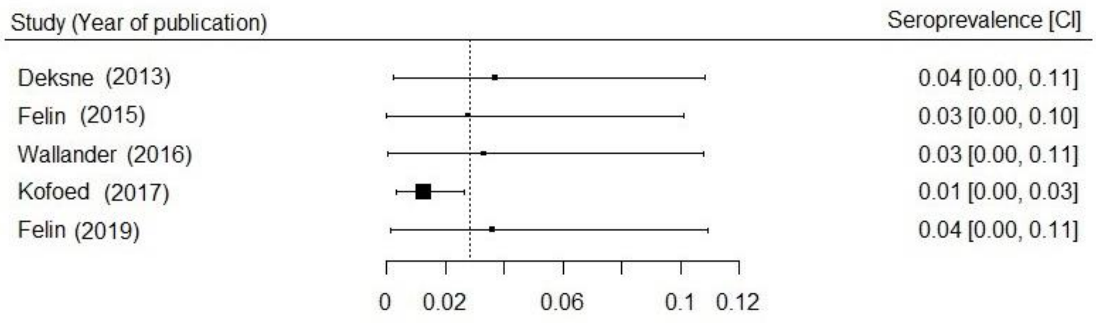

Figure B.14. Leave-one out analysis on studies reporting *Toxoplasma gondii* seroprevalence in domestic pigs raised in the indoor production system. Overall pooled seroprevalence for all five studies was 2.7 % ($CI_{95\%}$= 0–7, I^2= 96%). Each of the five pooled seroprevalence estimates calculated by leaving out each of the individual studies one by one were all well within the 95% confidence limits of the overall seroprevalence of all five studies.

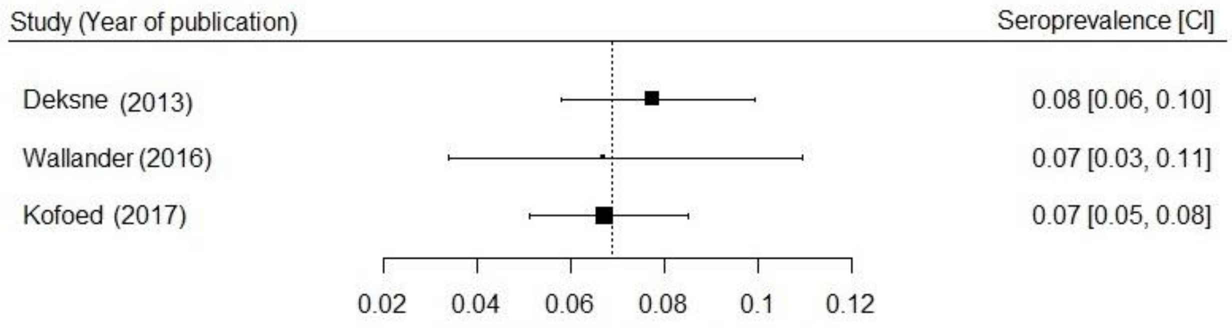

Figure B.15. Leave-one out analysis on studies reporting *Toxoplasma gondii* seroprevalence in domestic pigs raised in the outdoor production system. Overall pooled seroprevalence for all three studies was 7.9% ($CI_{95\%}$= 2–16, I^2= 98%). Each of the three pooled seroprevalence estimates calculated by leaving out each of the individual studies one by one were all well within the 95% confidence limits of the overall seroprevalence of all three studies.
