# Supplementary material for: Seroprevalence of Toxoplasma gondii in domestic pigs, sheep, cattle, wild boars, and moose in the Nordic-Baltic region: A systematic review and meta-analysis
Source: Parasite Epidemiol Control. 2019 Mar 4;5:e00100. doi: 10.1016/j.parepi.2019.e00100 (PMC6411595; doi:10.1016/j.parepi.2019.e00100)
Supplement: Appendix E — Instructions to the authors for the data-extraction sheet. [file mmc5.pdf]

Appendix E: Instructions to the authors for the data-extraction sheet

Column	Variable	Explanation
A	Author	Write the last name of the first author
B	Publication year	Select the year when the study was published in the journal and as mentioned in the article. For theses, conference abstracts, bulletins, reports take the date from the respective source
C	Article type	Select the type of publication. A secondary publication is one with no original data; e.g. from book, report, etc.
D	Country	Select the country where samples were collected. If the study is limited to a region of a country, then please specify the region(s) important in driving the seroprevalence in the comments field (column AC)
E	Start of sample collection (Year)	Select the year when the sample collection started
F	End of sample collection (Year)	Select the year when the sample collection ended
G	Species	Select the host species
H	Total number of animals (N)	Total number of animals included in the study.
I	Total number of seropositive animals (n)	Total number of animals that were *T. gondii* seropositive
J	Total number of animals kept exclusively indoors	Write the total number of sampled animals that were kept in indoor housing systems

K	Total number of seropositive animals kept exclusively indoors	Write the total number of sampled animals that were from indoor housing system and were *T. gondii* seropositive
L	Total number of animals that were or had access outdoors	Write the total number of sampled animals that were or had access outdoors
M	Total number of seropositive animals that were or had access outdoors	Write the total number of animals that were or had access outdoors and were *T. gondii* seropositive
N	Age of the animals reported in the study	Is the age of the animals included in the study reported? (Yes/No)
O	Total number of animals ≤ 1 year age	Write the total number of animals that belong to ≤ 1 year age group
P	Total number of seropositive animals ≤ 1 year age	Write the total number of *T. gondii* seropositive animals that belong to ≤ 1 year age group
Q	Total number of animals > 1 year age	Write the total number of animals that belong to > 1 year age group
R	Total number of seropositive animals > 1 year age	Write the total number of *T. gondii* seropositive animals that belong to > 1 year age group
S	Total number of animals of unknown age	Write the total number animals of unknown age
T	Total number of seropositive animals of unknown age	Write the total number of *T. gondii* seropositive animals of unknown age
U	Sample type	Select sample type
V	Serological test type	Select the type of the serologic test that was used. If

		the study used several tests, write a comment to column AE.<br><br>For in-house tests:<br><br>Tested = use of known positive or negative samples as controls AND/OR a commercial method reported<br><br>Not tested: use of known positive or negative samples as controls AND a commercial method NOT reported
W	Serological test trade name (if commercial test)	Leave empty if in-house test.
X	Serological test Sensitivity	Write test sensitivity, if it is reported. If not reported, write 'NO DATA'.
Y	Serological test Specificity	Write test specificity, if it is reported. If not reported, write 'NO DATA'.
Z	Test sensitivity & specificity discussed	Are sensitivity and specificity of the test discussed? (Yes/No)
AA	Cut-off value used	Write the cut-off for seropositivity used, if reported. If not reported, write 'NO DATA'.
AB	Comments about sampling: geographical area(s), number of farms, other aspects	Write description of the geographical area(s) the tested animals came from, number of farms included, and other aspects of the sampling that could affect the interpretation of the seroprevalence
AC	Other comments, in particular anything that could affect the interpretation of the seroprevalence	Write any further comments here.

General advice:
If the data is not provided, please write ‘NO DATA’
If some data are challenging to extract, please just leave a comment to that cell, and a designated team will check that.
